# Editorial: Metabolomics and the exposome

**DOI:** 10.3389/fpubh.2023.1188673

**Published:** 2023-06-28

**Authors:** Alexandros P. Siskos, Hector C. Keun, Benedikt Warth, Rachel S. Kelly, Lea Maitre

**Affiliations:** ^1^Cancer Metabolism & Systems Toxicology Group, Division of Cancer, Department of Surgery & Cancer and Division of Systems Medicine, Department of Metabolism, Digestion & Reproduction, Imperial College London, Hammersmith Hospital Campus, London, United Kingdom; ^2^Department of Food Chemistry and Toxicology, Faculty of Chemistry, University of Vienna, Vienna, Austria; ^3^Brigham and Women's Hospital and Harvard Medical School, Boston, MA, United States; ^4^Instituto Salud Global Barcelona (ISGlobal), Barcelona, Spain

**Keywords:** environmental health, exposomics, xenobiotics, biomarkers, environmental exposures, metabolomics, exposome

The human exposome represents the totality of all environmental exposures over a lifetime, from conception to death, and the effect of these exposures on human health and disease (1, 2). In recent years metabolomics has become a powerful tool to characterize environmental exposures and the human response to these exposures at the molecular level. Metabolomics can play an important role in understanding the link between environmental exposure and human health, contributing to the elucidation of disease etiology, identifying routes of exposure, and discovery of new biomarkers (3, 4). Moreover, metabolomics can potentially identify population groups that may be more susceptible to adverse health outcomes as a result of exposure. The study of the exposome and metabolomics is therefore becoming an integrated part of environmental health research that seeks to inform policymakers in ways to prevent or mitigate the effects of environmental exposure and pollution on a population level.

In this Research Topic, we invited original research, commentaries, and opinions to demonstrate the multidisciplinary nature of exposome research and the challenges in the use of metabolomics for this endeavor. Here, we highlight some of these aspects with emphasis on methodological and technological developments, data processing approaches, and the role of environmental, dietary, and occupational exposures.

One of the greatest challenges of metabolomics and exposome research is metabolite identification and the lack of suitable metabolite libraries and databases for this purpose. In the field of GC-MS, EI mass spectra have been compiled in libraries for years, however, most of these spectra have been reported at low resolution. Price et al., aiming to address the lack of high-resolution electron ionization mass spectral libraries (HR-[EI+]-MS) for environmental chemicals, constructed a retention-indexed HR-[EI+]-MS library following analysis of authentic compounds via GC-Orbitrap MS. The library is freely provided alongside a compound database of predicted physicochemical properties and offers a significant resource to the research community as an open science and open-source tool. Currently, the library contains over 350 compounds from 56 compound classes and includes a range of legacy and emerging contaminants. The RECETOX Exposome HR-[EI+]-MS library expands the number of freely available resources for use in full-scan chemical exposure studies and is available as described in the manuscript.

Metabolomics offers unique opportunities to identify food constituents and understand their role in the dietary exposome and food quality. In the manuscript by Nikou et al., the authors used state-of-the-art metabolomics methodologies to identify chemical biomarkers characteristic of geographical origin, cultivation practice, and production procedure of olive oil, a part of the Mediterranean diet that has been characterized as a healthy dietary pattern. Metabolomic profiling with Flow Injection Analysis - Magnetic Resonance Mass Spectrometry (FIA-MRMS) and LC-Orbitrap MS platform were used to analyze intact oil and the corresponding polyphenols of Extra Virgin Olive Oils. The methodology has the potential to offer a tool to tackle food fraud and adulteration and to also identify components of healthy dietary patterns.

An important part of the exposome is occupational exposures and patterns, including shift work, which in recent years has been associated with several physiological changes and potentially correlating with several diseases. In the manuscript by Borroni et al., the authors evaluated the effect of night shift work on the serum metabolome in a population of Italian female nurses working on night shifts and female colleagues not employed on night shifts, demonstrating differences in the levels of taurine, serotonin, aspartic acid, and some lipids, all of which offer clues on the biological alterations involved in shift work.

In the manuscript by Barupal et al., the authors demonstrated that the re-processing of metabolomics datasets, already published and publicly available, can change the number of metabolite identifications made and potentially uncover new biomarkers that may have been missed during the initial processing. The authors used MS-Dial for data pre-processing, a publicly available software, and applied less stringent data processing thresholds compared with the original data pre-processing approach that used the mass spectrometer manufacturer's software. The manuscript demonstrated the opportunities and also the challenges that arise from the fact that different software and even slight changes in pre-processing parameters can impact the quality and volume of data reported. The manuscript initiated a lively debate with two further commentaries by Keski-Rahkonen et al. and Barupal, which contributed to the overall metabolomics community discussions about methodologies, software and parameters of data pre-processing, reporting standards and formats, and inter-comparability between data pre-processing methodologies.

The study of the exposome and metabolomics is remarkably multidisciplinary and fast-moving, and this Research Topic captured some of the recent exciting developments and concepts in the field. We believe that the reader will find this Research Topic to be a useful reference that can inspire future research and innovations.

## Author contributions

This manuscript was initially written by AS. Manuscript was revised and edited by HK, RK, BW, and LM. All authors contributed to the article and approved the final submitted version.

## References

[1] Wild CP Scalbert A Herceg Z. Measuring the exposome: a powerful basis for evaluating environmental exposures and cancer risk. Environ Mol Mutagen. (2013) 54:480–99. 10.1002/em.21777 23681765

[2] Krausová M Braun D Buerki-Thurnherr T Gundacker C Schernhammer E Wisgrill L. Understanding the chemical exposome during fetal development and early childhood: a review. Annu Rev Pharmacol Toxicol. (2023) 63:517–40. 10.1146/annurev-pharmtox-051922-113350 36202091

[3] Vermeulen R Schymanski EL Barabási AL Miller GW. The exposome and health: where chemistry meets biology. Science. (2020) 367:392–6. 10.1126/science.aay3164 31974245 PMC7227413

[4] Rappaport SM Barupal DK Wishart D Vineis P Scalbert A. The blood exposome and its role in discovering causes of disease. Environ Health Perspect. (2014) 122:769–74. 10.1289/ehp.1308015 24659601 PMC4123034

